# Atypical epigenetic mark in an atypical location: cytosine methylation at asymmetric (CNN) sites within the body of a non-repetitive tomato gene

**DOI:** 10.1186/1471-2229-11-94

**Published:** 2011-05-20

**Authors:** Rodrigo M González, Martiniano M Ricardi, Norberto D Iusem

**Affiliations:** 1 Departamento de Fisiología, Biología Molecular y Celular, Facultad de Ciencias Exactas y Naturales, Universidad de Buenos Aires e IFIByNE-CONICET, Buenos Aires, Argentina

**Keywords:** epigenetics, asymmetric methylation, *Asr1*, water stress, tomato

## Abstract

**Background:**

Eukaryotic DNA methylation is one of the most studied epigenetic processes, as it results in a direct and heritable covalent modification triggered by external stimuli. In contrast to mammals, plant DNA methylation, which is stimulated by external cues exemplified by various abiotic types of stress, is often found not only at CG sites but also at CNG (N denoting A, C or T) and CNN (asymmetric) sites. A genome-wide analysis of DNA methylation in *Arabidopsis *has shown that CNN methylation is preferentially concentrated in transposon genes and non-coding repetitive elements. We are particularly interested in investigating the epigenetics of plant species with larger and more complex genomes than *Arabidopsis*, particularly with regards to the associated alterations elicited by abiotic stress.

**Results:**

We describe the existence of CNN-methylated epialleles that span *Asr1*, a non-transposon, protein-coding gene from tomato plants that lacks an orthologous counterpart in *Arabidopsis*. In addition, to test the hypothesis of a link between epigenetics modifications and the adaptation of crop plants to abiotic stress, we exhaustively explored the cytosine methylation status in leaf *Asr1 *DNA, a model gene in our system, resulting from water-deficit stress conditions imposed on tomato plants. We found that drought conditions brought about removal of methyl marks at approximately 75 of the 110 asymmetric (CNN) sites analysed, concomitantly with a decrease of the repressive H3K27me3 epigenetic mark and a large induction of expression at the RNA level. When pinpointing those sites, we observed that demethylation occurred mostly in the intronic region.

**Conclusions:**

These results demonstrate a novel genomic distribution of CNN methylation, namely in the transcribed region of a protein-coding, non-repetitive gene, and the changes in those epigenetic marks that are caused by water stress. These findings may represent a general mechanism for the acquisition of new epialleles in somatic cells, which are pivotal for regulating gene expression in plants.

## Background

Epigenetics refers to mitotically and meiotically heritable variation in gene regulation and function that cannot be accounted for by changes in DNA sequence but rather results from enzyme-mediated chemical modifications to DNA and its associated chromatin proteins [1]. Over the last decade, epigenetic research has focused mainly on mammals, whereas plants have received less attention, although there is a fair amount of information on certain plant models such as *Arabidopsis *[2,3], rice [4] and maize [5].

Whereas methylation in animal genomes occurs mostly in regulatory regions, methylation in *Arabidopsis *is found in transcribed sequences, not only at canonical CG sites but also at CNG (N denotes A, C or T) and CNN (asymmetric) sites. The latter sites are preferentially methylated in repetitive elements and transposons [6,7].

It has been well established through chemical analyses on mutants that MET1, the orthologous enzyme to mammalian DNMT1 (DNA methyltransferase 1), maintains DNA methylation at CG sites [8]. On the other hand, the plant-specific methyltransferase CMT3 maintains DNA methylation at CNG sites [7] while at the same time cross-talking with the histone H3 methyltransferase KYP [9]. Finally, the third type of plant cytosine methylation (CNN, called "asymmetric") was demonstrated by pioneer mutant analysis to arise due to the methylase DRM2 [10], a homologue of the mammalian *de novo *methyltransferase DNMT3. DRM2, together with endogenous small interfering RNAs, also maintains DNA methylation at CNN sites [11], a less-studied epigenetic modification.

Our studies focused on the tomato plant (*Solanum lycopersicum*), an edible plant crop (http://mips.helmholtz-muenchen.de/plant/tomato/index.jsp) of great economic importance with a genome that is almost 10 times larger than that of *Arabidopsis *and of which there have been few epigenetics studies [12]. Using this model system, we investigated cytosine methylation status in different contexts and the intragenic distribution of cytosine methylation in *Asr1*, a non-transposon, protein-coding, water stress-inducible gene of the LEA superfamily [13] that is conserved in the plant kingdom but lacks an orthologous counterpart in *Arabidopsis*. This gene has been extensively studied by us and other groups at the DNA [14], RNA [15] and protein [16,17] levels and in terms of physiological function [18] and evolution [14]. This 1,199-bp gene has a very simple organisation, consisting of exon 1 and exon 2 of 153 and 358 nt, respectively, separated by an intron of 688 nt. We chose the leaf as the source of genomic DNA because it is the organ in which *Asr1 *expression is the greatest upon water stress [15].

A second aspect of our work dealt with the intriguing link between epigenetics and stress in plants [19-21]. Stress-induced physiological responses in *Arabidopsis *are thought to depend on altered DNA methylation [22]. To test this hypothesis experimentally, we examined the gain and loss of cytosine methylation marks on our model gene as a consequence of imposing water stress conditions on tomato plants.

## Results

### Overall non-CG methylation in the tomato genome

**Figure 1:**
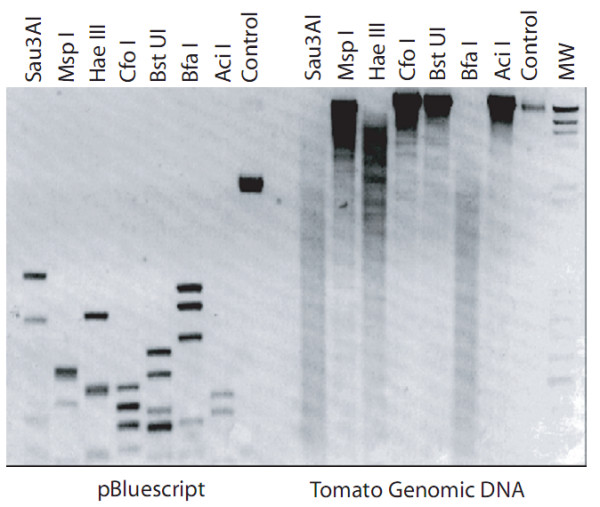
**Panoramic view of CG and CNG methylation in the tomato plant**. Total leaf genomic DNA was treated with the indicated restriction enzymes (right). Recognition sites are listed in the Methods section. As a control for enzymatic cutting efficiency and specificity, pBluescript plasmid was similarly treated (left).

To explore the general features of methylation in tomato leaf DNA, we first observed a panoramic view of both CG and CNG methylation using several restriction enzymes. Comparisons between methylation-sensitive and -insensitive enzymes provided an evaluation of the overall CG methylation. This low-resolution but illustrative analysis (Figure 1) displayed a pronounced level of typical CG methylation and a noticeable degree of overall CNG methylation (Figure 1, Msp I treatment), a modification that is typically, though not exclusively, associated with repeated and/or transposable elements.

### Non-CG methylation in the *Asr1 *gene body

Motivated by the results described above, we wanted to gain insight into methylation events in cytosine contexts other than the well-known CpG. For that purpose, we performed a closer inspection of *Asr1 *in the leaf.

**Figure 2:**
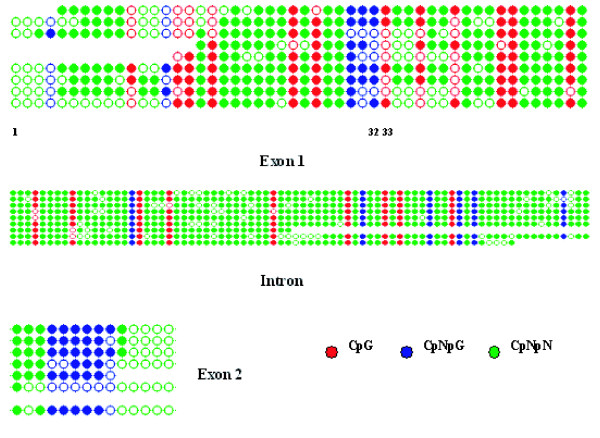
***Asr1 *basal methylation status**. Leaf genomic DNA was subjected to the bisulphite procedure and then cloned (9 independent clones) and sequenced. Results are displayed as dot plots (Kismeth software) as described in the Methods section. Filled circles, methylated; empty circles, unmethylated. The numbers indicate cytosine positions beginning from the first analysed cytosine. Residues 32 and 33 are particular cytosine residues that were individually analyzed later (Figure 6.).

**Figure 3:**
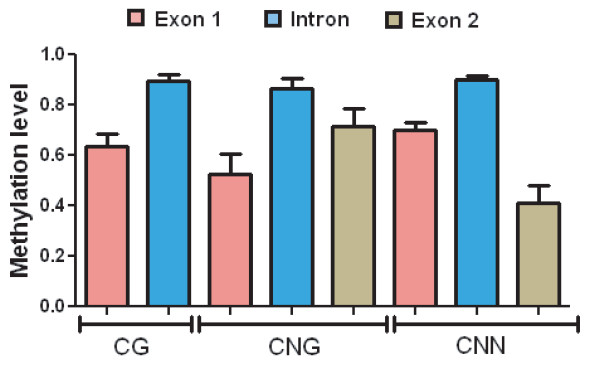
**Survey of *Asr1 *basal methylation levels**. Data from Figure 2 were grouped by methylation type and gene region.

For this analysis, we used the bisulphite procedure [23], which allows a higher resolution as it is able to detect all cytosine residues (Figure 2), not just the residues within a site recognised by a restriction enzyme. After pooling the data for each methylation type and grouping by gene region (Figure 3), we concluded that there are significant levels of the three types of methylation (CG, CNG and CNN) under non-water stress conditions. We were surprised to detect CNN, as *Asr1 *is a non-transposon gene bearing no repetitive elements and hence constitutes a novel location for this type of methylation site. In this case, CNN turned out to be concentrated preferentially in the intron.

To address methodological concerns, we performed parallel bisulphite reactions on non-methylated or in vitro methylated plasmid DNA and obtained expected outcomes (see Methods). In addition, we ruled out unintentional overestimation of cytosine methylation due to an eventual inefficient bisulphite conversion by using primers that were specifically designed to amplify the converted template and were incapable of annealing to the natural template. Furthermore, there is no reason to believe that some cytosine residues (the ones complementary to the primers) were in fact converted while others in the same pure DNA sample were not.

### CNN demethylation upon water stress

**Figure 4:**
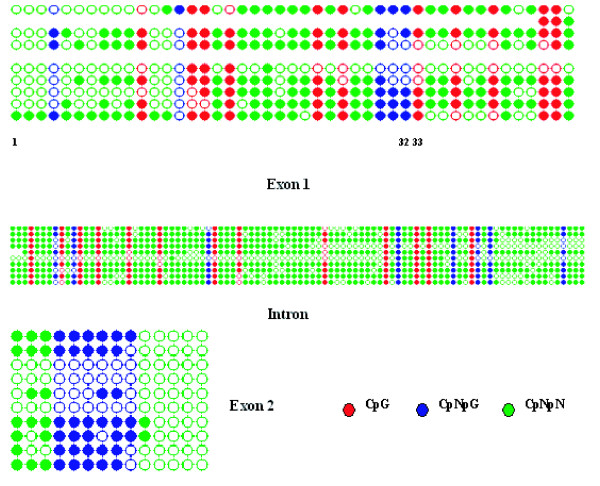
***Asr1 *methylation status following stress**. Leaf genomic DNA from water-stressed plants was subjected to the bisulphite procedure and then cloned (10 idependent clones) and sequenced. Results are displayed as dot plots (Kismeth software) as described in the Methods section. Filled circles, methylated; empty circles, unmethylated. The numbers indicate cytosine residue positions starting from the first analysed cytosine. Residues 32 and 33 are particular cytosine residues that were individually analysed later (Figure 6.).

**Figure 5:**
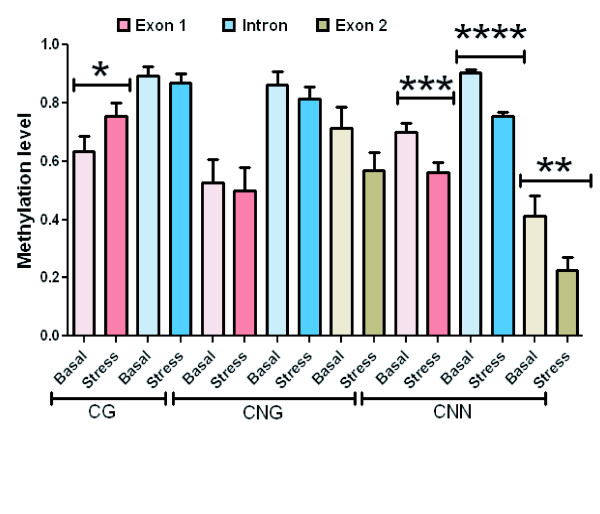
**Survey of *Asr1 *methylation levels in normal and stressed plants**. Data from Figures 2 and 4 were grouped by methylation type and gene region. *p < 0.08; ** p < 0.05; *** p < 0.005; **** p < 0.0001.

To understand the molecular mechanisms underlying the adaptation of plants to abiotic stress, we tested the hypothesis that stress-induced phenotypes depend on epigenetic changes. With that goal in mind, a similar type of experimental analysis was performed on leaf DNA after imposing water-shortage stress on whole tomato plants through root drying. We found that drought-simulated conditions brought about methylation at CG sites in exon 1 (p < 0.08) and simultaneous removal of methyl marks at 75 of the 110 asymmetric (CNN) sites analysed. This demethylation scenario was statistically significant throughout the gene body as follows: exon 1 (p < 0.005), intron (p < 0.0001) and exon 2 (p < 0.05) (Figures 4 and 5).

These results are in agreement with the methylation status data obtained by direct (i.e., with no previous subcloning) sequencing of the *Asr1 *PCR product after bisulphite treatment of the genomic DNA (data not shown).

It is worth noting that clones with dissimilar patterns may have arisen from different cell types (e.g., epidermis and guard cells) together in the leaf samples under examination, each displaying a distinct epigenetic behaviour.

**Figure 6:**
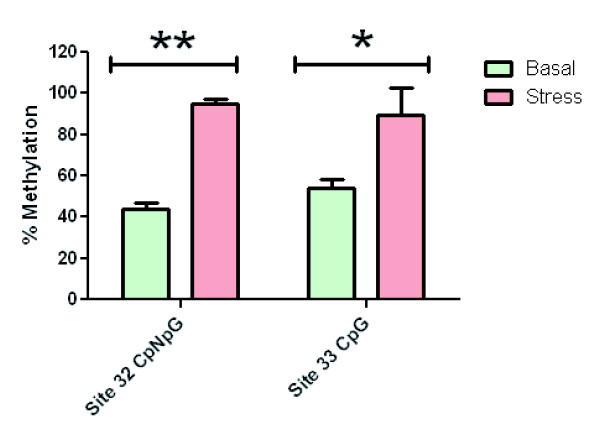
**Analysis of methylation at a particular site**. A pair of isoschizomers (HpaII and MspI) with different methylation specificities was used as described in the Methods section to discriminate between CG and CNG contexts in the leaves of both normal and stressed plants. For site 32 (indicative of CNG methylation), **p < 0.0001; for site 33 (indicative of CG methylation), *p < 0.01)

With the intention of further validating the bisulphite methodology, we measured the extent of methylation at a single CCGG site (which obviously contains both CG and CNG contexts) by methylation-sensitive and -insensitive restriction enzymes; the chosen site was C_32_C_33_GG, belonging to exon 1. The result (Figure 6) is in agreement with that obtained with bisulphite for those particular cytosine residues for both basal and stress conditions (Figure 2 and 4).

At this point, it is pertinent to clarify that the methylation trends shown in Figures 3 and 5 reflect an average behaviour of all cytosine positions grouped in each gene region and thus may not necessarily match the epigenetic situation of individual cytosine residues like those depicted in Figure 6.

**Figure 7:**
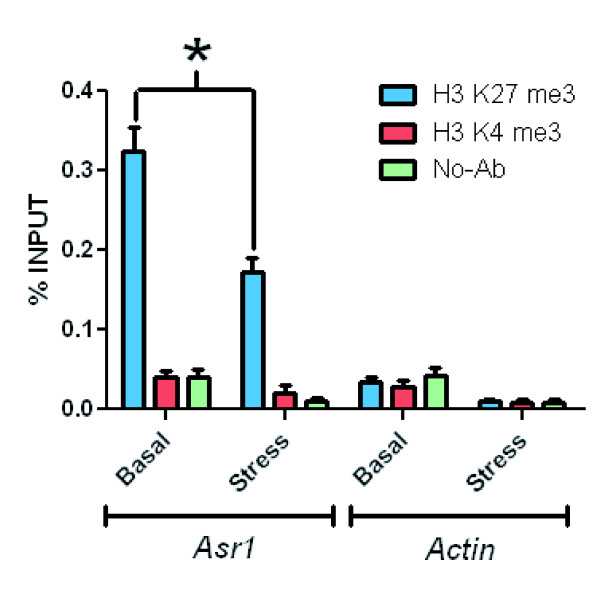
**Association of Asr1 with a repressive histone epigenetic mark**. ChIP was performed using Dynabeads protein A (Invitrogen) and anti-H3K4me3 or anti-H3K27me3 antibodies (Abcam). Quantitative Real-Time PCR was carried out as indicated in the Methods section. Comparison between non-stress vs. stress yielded p < 0.05. Actin was included as a housekeeping gene control.

As gene expression could be regulated also by post-translational histone modifications, which, in turn, may interact with the methylation of cytosines, we decided to explore H3K27me3 and H3K4me3, abundant histone marks in *Arabidopsis *[24]. We found the expression level of gene Asr1 tightly associated with H3K27me3, a major repressive mark for gene expression. Such a covalent modification quantitatively appeared to decrease with water stress (p < 0.05) (Figure 7). In contrast, H3K4me3, a mark distinctive of gene activation, was not significantly detected under any condition in the context of *Asr1 *(Figure 7).

### Asr induction upon water stress

**Figure 8:**
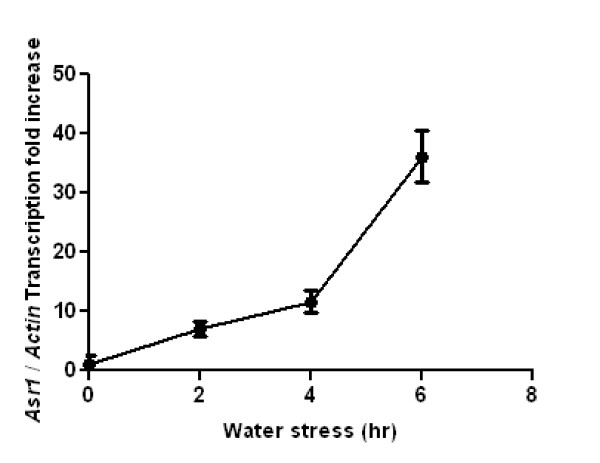
***Asr1 *is induced upon stress**. Water-stress time course of *Asr1 *leaf mRNA steady-state levels quantified by real-time RT-PCR. Actin mRNA (considered a constitutive transcript) was measured at each time and hence served as a loading control for normalisation purposes.

To identify an eventual correlation between any type of methylation (CG, CNG and CNN) and expression of our model gene, we performed qRT-PCR for both basal and stress conditions. The results (Figure 8) indicate a 7-fold induction of *Asr1 *leaf mRNA levels after 2 hours of water stress, reaching a robust 36-fold induction at 6 hours, the time point at which the marked wilting phenotypes observed in the roots and leaves were still reversible (data not shown).

## Discussion

The typical CG methylation within promoter regions observed in animal genomes has also been recognised in certain plant loci [25]. However, epigenome-wide surveys in *Arabidopsis *have revealed that transcribed regions are also capable of being methylated, but to a lesser extent compared to transposons, and methylation is limited to CG sites [26]. One such example comes from a study with petunia showing that a class-C floral homeotic gene was expressed following transgene-induced RNA-directed DNA methylation (RdDM) at CG sites in an intron [27], which also revealed that DNA methylation in gene bodies is not necessarily associated with silencing as it is in animals. Another similar example was reported by Zhang et al. [28], who found that many housekeeping genes were methylated in coding regions and actually showed a higher level of expression. In accordance with these data, we found stress-provoked higher CG methylation levels in the first exon of our model gene, concomitantly with enhanced gene expression.

On the other hand, evidence of non-CG methylation in tandem repeats has been accrued by the Jacobsen group [29] along with its conservation across duplicated regions of the genome [30]. In our work, we detected extensive asymmetric CNN methylation in a novel location: a non-repeat transcribed region. In addition, we found that such an epigenetic modification correlated with poor expression, consistent with older work [31]. Similarly, a null DRM2 mutant was reported to block non-CG methylation, which allowed for full desilencing of the FWA gene, resulting in a late-flowering phenotype [32].

Current models propose that methyl-cytosine-binding proteins, through their SRA (SET and RING-associated) domains, link DNA and histone methylation events [33]. Indeed, DNA methylation can induce chromatin remodelling by recruiting methylcytosine-binding proteins such as KYP, a H3K9 methyltransferase, and VIM1, which in turn induce heterochromatinisation [34]. Self-enforcement of CNN methylation by DRM2 is also mediated by SUVH9, which has no detectable histone methyltransferase activity but binds methylated CNN sites, thus facilitating further access for DRM2 to methylated regions [35].

It seems significant that cytosine methylation in the bodies of protein-coding genes may be lost at high frequency in successive generations [36], which is in agreement with our results showing a heterogeneous population of epialleles in basal conditions. A similar scenario of variation has also been found for naturally repeated RNA genes [37].

Interestingly, intragenic DNA methylation mechanisms are emerging as essential modifications, as they regulate gene expression and plant development [1], but how those mechanisms operate remains an important question. One example of the existence of additional molecular players is provided by genetic evidence that a particular *Arabidopsis *mutant undergoes ectopic deposition of CNG methylation in thousands of genes [38]. These data suggest that there is a set of as-yet-unexplored, genome-protecting factors that play a role in blocking methyltransferases from modifying gene regions containing non-CG sites that may include CNN sites.

At this point, it is worth mentioning that, to the best of our knowledge, conclusions as to the assignment of different plant methylases to particular substrate sites have been derived solely through reverse genetics by analysing mutants [7,10,39] and not from in vitro experiments with purified enzymes. A biochemical approach to do so does not yet exist but, if developed, would convincingly validate current hypotheses. Moreover, biochemistry would help to elaborate new models needed to understand the in vivo maintenance of CNN methylation during DNA replication, which is difficult to envisage, as there are no local cytosine residues to be methylated in the nascent complementary strand.

As far as the appealing connection between plant epigenetics and stress is concerned, our findings in the tomato plant are consistent with the hypothesis highlighted by the Kovalchuk group [22] in *Arabidopsis*, and experimentally supported in rice [40], that at least some stress-induced phenotypes depend on altered DNA methylation.

Regarding chromatin architecture, it is not surprising that H3K27me3 resulted in association with the expression level of gene Asr1 in basal conditions rather than under stress, since it is a major repressive mark, at least in *Arabidopsis *[24].

In conclusion, the data presented here show a novel location for CNN methylation in plants, namely in the body of a model gene with no repeated sequences that is regulated by water stress. These findings may represent an alternative and general mechanism for the stress-driven gain or loss of epigenetic marks that regulate gene expression in plants other than *Arabidopsis*, which have larger and more complex genomes. The rapid appearance of these newly acquired epialleles in the affected somatic cells, coupled with the unique ability of plants to produce germline cells late during development, may allow its inheritance across generations [41,42] and eventual positive selection, thus contributing to adaptive evolution.

## Conclusions

1) There is a noticeable degree of overall CNG methylation in the *Solanum lycopersicum *genome, a modification that is typically, though not exclusively, associated with repeated and/or transposable elements.

2) We found a heterogeneous population of epialleles in the *Asr1 *gene under both basal and water stress conditions.

3) We detected an extensive asymmetric CNN methylation in a novel location: a transcribed region of a protein-coding, non-repetitive gene, correlating with poor expression.

4) Drought conditions brought about higher CG methylation levels in the first exon of our model gene and removal of methyl marks at CNN sites, mostly in the intronic region, concomitantly with enhanced expression of this gene.

5) Drought conditions caused a decrease of H3K27me3 in the context of our model gene, concurrently with enhanced expression of this gene.

## Methods

### Plant material

Commercial tomato (*Solanum lycopersicum*) seeds were bleached by sinking in a 20 g/l sodium hypochlorite solution for 30 min. After the treatment, the seeds were placed on dampened blotting paper and left in the dark for 72 hr. Plantlets were placed in a growth chamber at 23°C with a photoperiod of 12 hr light/12 hr dark for 5 days followed by transplantation to pots. Plants were then returned to the growth chamber and watered twice a week until experiments were performed.

### Water stress conditions

Four 3-week-old plants were taken from the pots, and their roots were carefully cleaned. Leaves from two plants were cut off and frozen in liquid nitrogen (non-stressed plants). From the two other plants, roots were put on blotting paper under an incandescent lamp for approximately 2 hr until a wilting phenotype (proved to be reversible) appeared. Leaves were cut off and immediately frozen (stressed plants).

### DNA extraction

Peralta and Spooner's protocol [43] was followed with some modifications. This procedure includes the use of CTAB as a detergent instead of SDS, which is appropriate for the tomato plant due to its high content of sugars and polyphenols. DNA quality was assessed by spectrophotometry by means of the A_260_/A_280 _ratio. Only samples with A_260_/A_280 _ratio between 1.7 and 2.0 were used.

### Estimation of overall genome methylation by means of restriction enzymes

Total genomic DNA (100 ng) was treated with restriction enzymes that exhibit distinct sensitivity to cytosine methylation (Table 1). Incubations were carried out in 20 μl (final volume) with 5 U enzyme at 37°C for 4 hr in all cases.

**Table 1:** Recognition sites and sensitivities to methylation inherent to the restriction enzymes used in the experiment shown in Figure 1.

Enzyme	Recognition site	Sensitivity to methylation
Aci I	5'-CCGC-3'	CpG

Bfa I	5'-CTAG-3'	none

BstU I	5'-CGCG-3'	CpG

Cfo I	5'-GCGC-3'	CpG

Hae III	5'-GGCC-3'	none

MspI	5'-CCGG-3'	CpNpG

Sau3A I	5'-GATC-3'	none

### Methylation analysis at specific sites by means of restriction enzymes

Inspection of methylation status was performed on a pair of contiguous cytosine located in exon 1 of *Asr1 *(GenBank accession number L08255) by mens of isoschizomers (HpaII and MspI; recognition site 5'-CCGG-3') that display different sensitivities to methylation depending on nucleotide context; whereas HpaII is sensitive to methylation at the internal cytosine (indicative of CpG methylation), MspI is sensitive at the external cytosine (probe for CpNpG methylation) [44].

After each enzymatic reaction, real-time PCR was performed to quantify the 169-bp amplicon generated from the following primers flanking the cutting site: forward primer 5'-ATGGAGGAGGAGAAACACC-3' and reverse primer 5'-GATTATATCAACGTACCAAGGC-3'. For PCR purposes, we used Taq DNA Polymerase (Invitrogen) (0,625 U), 3 μM MgCl_2_, 0.2 μM dNTPs and 0.2 μM of each primer. 5 ng (1 μl) of template was added. The final reaction volume was 25 μl. The equipment used was an MJ Engine Opticon (BioRad) with SybrGreen^® ^as the fluorophore under the following conditions: 40 cycles of denaturation (94°C, 30 sec), annealing (67°C, 30 sec) and elongation (72°C, 45 sec). Melting curve was made from 70 to 95°C every 0.5°C. All PCR reactions were run by duplicate and 4 non-template negative controls were included. We also made a standard curve to validate PCR linear range, sensitivity and limit of detection. For amplification data analysis, we used Opticon Monitor software, provided by PCR manufacturer. Plates and lids were provided by Axygen and oligonucleotides were purchased from IDT Inc.

The occurrence of near-full cutting due to the absence of methylation was inferred if late amplification of the long fragment was observed. Conversely, methylated, and hence uncut, DNA allowed early amplification of the long fragment under the same conditions. C(t) values were normalised to a non-relevant amplicon lacking the restriction site. For that purpose, we used an actin (GeneBank accession number AB199316.1) couple of primers (Forward: 5'-GGGATGATATGGAGAAGATATGG-3' and Reverse: 5'-AAGCACAGCCTGGATAGC-3') that amplifies an 185-bp amplicon, under the same cycling conditions and reagents concentrations. ΔC(t) values (enzyme-treated vs. untreated) were calculated for each enzyme: the greater the methylation, the lower the ΔC(t) value, and vice versa. In all cases the PCR product specificity was check by melting curve analysis and 2% agarose electrophoresis.

### Bisulphite procedure

We used the protocol described by Clark et al. [23] with some modifications. DNA was digested with Bfa I (5'-CTAG-3') at 37°C overnight to obtain DNA fragments of approximately 2,000 bp in average length, which were partially purified by extraction with phenol:chloroform (1:1). Total genomic DNA (1 μg) was then treated with bisulphite; the conversion step was performed for 16 hr at 55°C. Treated DNA was then purified using the commercial Wizard DNA Clean-Up System kit (Promega).

### Post-bisulphite PCR

The *Asr1 *gene (GenBank accession number L08255) was amplified using primers that were previously designed [45] with the highest C+G content possible to favour annealing to template and with the highest content of thymine residues derived from bisulphite-converted cytosine residues, especially in the 3' ends, which favour the selective amplification of converted molecules. Primers were designed using the Beacons Designer software (http://www.premierbiosoft.com/molecular_beacons/index.html).

Semi-nested regular PCR was chosen to minimise the risk of amplifying non-converted DNA. For the first reaction (1,044-bp amplicon), 5 μl of bisulphite-treated product was amplified by Taq DNA Polymerase (Invitrogen) in an MJ Research PTC-100 (MJ Research Inc.) according to the following program: 40 cycles of denaturation (94°C, 30 sec), annealing (50°C, 30 sec) and elongation (72°C, 1.30 min); PCR was performed using the following primers: forward primer 5'-ATAGAGGATTTGATAAGATTATATTTG-3' and reverse primer 5'-CTTTTTTCTCATAATACTCATAA-3'. For the second reaction, a forward primer, internal to the first one, was used, as follows: forward 5'-GGAGGAGGAGAAATATTATTATT-3'.

As a reverse primer, the same one was used under the same cycling conditions. The final amplicon obtained was 966 bp long, comprising the entire exon 1, the intron and the first 104 nt of exon 2. In both PCR reactions we used 0,625 U of Taq DNA polymerase, 6 μM MgCl_2_, 0.2 μM dNTPs and 0.2 μM of each primer in a final volume of 50 μl.

### Validation of bisulphite conversion efficiency

To assess the full conversion, plasmid DNA (pBluescript SK+, Stratagene) was first linearised and then methylated in vitro by the methyltransferase mHaeIII (5'-GGmCC-3') (New England Biolabs). Both enzyme-treated and untreated plasmids were incubated with bisulphite under the same conditions as the genomic DNA samples, followed by PCR with primers designed specifically for this experiment, as follows: forward primer 5'-TTGTTATTATGTTAGTTGGTGAAAGG-3' and reverse primer 5'-CCCAAACTTTACACTTTATACTTCC-3'.

The resulting 383-bp amplicon was incubated with BfaI (5'-CTAG-3'); when the plasmid was not previously bisulphite-treated, the enzyme cut the amplicon into two expected fragments of 212 and 171 bp, but when the plasmid was bisulphite-treated, the site was lost (now 5'-TTAG-3'), and the enzyme was not able to cut. Furthermore, when the plasmid was previously methylated by mHaeIII, the creation of the new site, because of modification of the sequence 5'-GGCCAG-3' to 5'-GGCTAG-3', was confirmed by gel detection of the two expected bands of 285 and 98 bp obtained after cutting.

### Subcloning and sequencing

Subcloning was performed in the pGEM-T "easy vector" (Promega). Plasmid minipreps were processed from randomly picked insert-positive colonies (10 for each biological situation) using the GeneJET Plasmid Miniprep Kit (Fermentas). Sanger sequencing was carried out from SP6 and T7 universal primers.

### Methylation data analysis

Kismeth software [46] (http://katahdin.mssm.edu/kismeth/revpage.pl) was used to analyse the methylation data. Once the data for each site were gathered, GraphPad software was used for statistical analysis. The data were grouped according to gene region (exon 1, intron, exon 2) and methylation type (CpG, CpNpG, CpNpN). Statistical analysis was performed using the Mann-Whitney test at the 95% significance level.

### Direct methylation analysis of post-bisulphite PCR products

PCR products were purified using gel electrophoresis and a QIAquick Gel Extraction Kit (Qiagen) and sequenced without previous subcloning using the same primers used for PCR. Chromatograms were analysed using VarDetect [47] to estimate the ratio of cytosine to thymine signal. Statistical analysis was performed using the Mann-Whitney test at the 95% significance level.

### Chromatin immunoprecipitation (ChIP) for histone modifications

We followed Ricardi *et al*'s protocol [48], but using Dynabeads protein A (Invitrogen) instead of agarose beads. We used 2 μg of DNA for input and 8 μg for every treatment. To keep those amounts constant, volumes were variable according to DNA initial concentrations. Anti-H3K4me3 and H3K27me3 antibodies were purchased from Abcam. Quantitative Real-Time PCR was performed using the same primers for *Asr1 *and actin, as used in the methylation analysis by means of restriction enzymes (Figure 6). We used the same cycling conditions and reagents concentrations as in the restriction enzyme experiment (Figure 6) but using Maxima Hot Start DNA polymerase (Fermentas).

### Expression analysis (RNA extraction, retrotranscription and qRT-PCR)

Total RNA was extracted with Trizol (Invitrogen) from 100 mg of mortar-grounded leaves in liquid nitrogen followed by incubation with 12.5 U DNAsaI (Invitrogen). Retrotranscription was achieved using 2 μl of RNA, 50 U MMLV-RT (Promega) and oligo-dT (50 pmoles) in a 25 μl final volume, for 1 hr at 42°C. To prevent RNA degradation, 10 U of RNAseOUT (Invitrogen) was added. Real-time PCR was performed under the same conditions indicated above, using the following primers:

*Asr1  337 bp* 5'-CAGATGGAGGAGGAGAAACAC-3'   5'-TAGAAGAGATGGTGGTGTCCC-3'

*Actin 185 bp* 5'-GGGATGATATGGAGAAGATATGG-3' 5'-AAGCACAGCCTGGATAGC-3'

Data obtained for *Asr1 *mRNA were normalised to actin mRNA at each stress time before comparing different stress treatments.

## Abbreviations

*Asr1: *Abcisic Acid Stress and Ripening 1; DNA: Deoxyribonucleic acid; RNA: Ribonucleic Acid; MET1: Methyltransferase 1; DNMT1: DNA methyltransferase 1; CMT3: Chromomethylase 3; KYP: Kryptonite Histone 3 Lysine 9 Methyltransferase; DRM2: Domains Rearranged Methyltransferase 2; LEA: Late Embryogenesis Abundant; PCR: Polymerase Chain Reaction; qRT-PCR: Quantitative Real Time - Polymerase Chain Reaction; FWA: Flowering Wageningen; VIM1: Variant in Methylation 1; SUVH9: SU (Var) 3-9 Homolog 9; CTAB: Cetyl Trimethyl Ammonium Bromide; SDS: Sodium Dodecyl Sulphate; RdDM: RNA-directed DNA methylation.

## Competing interests

The authors declare that they have no competing interests.

## Authors' contributions

RMG performed all experimental work, generated the data and extensively revised the manuscript together with NDI. MMR supported the daily lab tasks and made valuable suggestions throughout the work, particularly the ChIP experiments required for the revised version. NDI introduced the theoretical frame, coordinated the project and drafted the manuscript. All authors read and approved the final manuscript.

## Authors' information

RMG and MMR hold doctorate fellowships from Consejo Nacional de Investigaciones Científicas y Técnicas (CONICET), Argentina. NDI is an Independent Researcher of CONICET.
